# A Novel Peptide as a Specific and Selective Probe for *Klebsiella pneumoniae* Detection

**DOI:** 10.3390/bios12030153

**Published:** 2022-03-01

**Authors:** Hyun Kim, Ju Hye Jang, In Young Jung, Ju Hyun Cho

**Affiliations:** 1 Research Institute of Life Sciences, Gyeongsang National University, Jinju 52828, Korea; hyun.kim@gnu.ac.kr (H.K.); juhye.jang@gnu.ac.kr (J.H.J.); 2 Division of Applied Life Science (BK21Four), Gyeongsang National University, Jinju 52828, Korea; inyoung.jung@gnu.ac.kr; 3 Division of Life Science, Gyeongsang National University, Jinju 52828, Korea

**Keywords:** *Klebsiella pneumoniae*, phage display peptide, pathogen detection, lipopolysaccharide, selective probe

## Abstract

*Klebsiella pneumoniae* is infamous for generating hospital-acquired infections, many of which are difficult to treat due to the bacterium’s multidrug resistance. A sensitive and robust detection method of *K. pneumoniae* can help prevent a disease outbreak. Herein, we used *K. pneumoniae* cells as bait to screen a commercially available phage-displayed random peptide library for peptides that could be used to detect *K. pneumoniae*. The biopanning-derived peptide TSATKFMMNLSP, named KP peptide, displayed a high selectivity for the *K. pneumoniae* with low cross-reactivity to related Gram-negative bacteria. The specific interaction between KP peptide and *K. pneumoniae* lipopolysaccharide resulted in the peptide’s selectivity against *K. pneumoniae*. Quantitative analysis of this interaction by enzyme-linked immunosorbent assay revealed that the KP peptide possessed higher specificity and sensitivity toward *K. pneumoniae* than commercially available anti-*Klebsiella* spp. antibodies and could detect *K. pneumoniae* at a detection limit of 10^4^ CFU/mL. These results suggest that KP peptide can be a promising alternative to antibodies in developing a biosensor system for *K. pneumoniae* detection.

## 1. Introduction

Hospital-acquired infections (HAIs) are considered a public health concern because they increase mortality and morbidity, lengthen hospitalization, and result in high healthcare costs [1]. A leading cause of these HAIs is *Klebsiella pneumoniae*, a member of the *Enterobacteriaceae* family that often causes pneumonia, bacteremia, pyogenic liver abscesses, and urinary tract infections, with the majority of these infections occurring in immunocompromised patients [2]. *K. pneumoniae* colonizes human mucosal surfaces easily, including the gastrointestinal tract and oropharynx, where its effects appear to be benign [2,3,4]. *K. pneumoniae* can enter other tissues from these sites and cause severe infections. Epidemiological data indicate that many hospitalized patients have *K. pneumoniae* in their gastrointestinal tract, linking *K. pneumoniae* carriage and subsequent disease from the same isolate [5,6,7]. Individuals can be silently colonized for extended periods, and these asymptomatic carriers serve as reservoirs for persistent transmission, making spread challenging to control and outbreaks challenging to stop [8,9,10]. Moreover, *K. pneumoniae* infections acquired in hospitals are challenging to treat since many *K. pneumoniae* strains have become highly drug-resistant [11,12]. Hence, this bacterium’s rapid, precise, and sensitive identification is required to guide the appropriate therapy and control the pathogen’s spread.

Microscopic examination, biochemical identification, and automatic bacterial detection apparatus such as the VITEK 2 system are traditionally used to detect *K. pneumoniae* based on the phenotypic system. However, they have low sensitivity and are time-consuming due to requiring several days of incubation [13]. PCR-based assays (e.g., conventional PCR, multiplex PCR, or real-time PCR) provide high sensitivity and specificity and have become the gold standard for detecting *K. pneumoniae* [14,15,16]. However, PCR-based assays require expensive equipment, trained personnel, and a consistent power supply, making them unsuitable for on-site testing. Recently, several investigators have developed alternative methods, including MALDI-TOF MS [17,18], luminescent phage [19], gold nanoparticle (GNP) [20], and lateral-flow strip immunoassay (LFSA) [21] for *K. pneumoniae* detection. However, these methods also have drawbacks such as the need for sophisticated equipment (MALDI-TOF MS), the need for a relatively long incubation time (luminescent phage), and low sensitivity (GNP and LFSA).

Biosensors are a sophisticated alternative to commonly used molecular bacterial detection methods, providing cost-effective, specific, sensitive, and in situ real-time monitoring with minimal sample preparation and detection time [22]. Biosensors are essentially made up of a recognition element coupled to a transducer that converts specific analyte binding to receptors into a detectable or measurable readout [23,24]. Therefore, developing biosensors with high sensitivity and specificity requires the design and use of recognition elements that specifically bind to the pathogen of interest. Although antibodies are the most commonly used recognition element in affinity biosensor research, their production and purification costs, as well as their stability during and after immobilization on sensor surfaces, are difficult to manage [24]. Short peptides derived from phage-displayed libraries have recently been introduced as an intriguing alternative for developing novel biosensing platforms. As opposed to antibodies, peptides are more stable and easier to manipulate at the molecular level, and they have a low manufacturing cost [25,26]. In fact, phage-displayed peptides have been successfully employed as molecular recognition elements for the detection of bacterial toxins [27] as well as the identification of *Staphylococcus aureus* [28,29], *Pseudomonas aeruginosa* [30], *Salmonella typhimurium* [31,32], and *Bacillus anthracis* [33].

In this study, we used a phage display approach to isolate peptides that bind selectively to the surface of *K. pneumoniae*. The ability of the isolated peptide to interact specifically with *K. pneumoniae* was assessed using enzyme-linked immunosorbent assay (ELISA) and confocal laser scanning microscopy by comparing it to a panel of other bacterial species. The potential target for the isolated peptide on the *K. pneumoniae* surface was identified. In addition, the specificity and sensitivity of the isolated peptide for detecting *K. pneumoniae* were further evaluated in comparison with commercially available anti-*Klebsiella* spp. antibodies.

## 2. Materials and Methods

### 2.1. Bacterial Strains and Culture

The bacterial strains used in this study were obtained from the American Type Culture Collection (ATCC) and the Korean Collection for Type Culture (KCTC) and comprised: *K. pneumoniae* KCTC 2208, *Escherichia coli* KCTC 2223, *P. aeruginosa* ATCC 27853, *Pseudomonas putida* ATCC 17426, *S. typhimurium* KCTC 2370, *Salmonella enteritidis* ATCC 13076, *S. aureus* KCTC 1621, *Staphylococcus epidermidis* KCTC 1917, *Enterococcus faecalis* KCTC 2011, *Bacillus subtilis* ATCC 6633, and *Lactococcus lactis* ATCC 11454. Clinically isolated *K. pneumoniae* strains (KBN 12P00150, KBN 12P00173, KBN 12P00237, and KBN 12P02404) and antibiotic-resistant *K. pneumoniae* strains [NCCP 15782 (NDM-1), NCCP 16124 (GES-5), and NCCP 16128 (OXA-232)] were obtained from the Gyeongsang National University Hospital Culture Collection for Pathogens and National Culture Collections for Pathogens, respectively.

Cultures were maintained and subcultured on nutrient agar plates on a regular basis and stored at 4 °C until tested. Bacterial stock cultures were stored at −80 °C in tryptic soy broth (TSB) or M17 broth supplemented with 0.5% glucose for *L. lactis* with 20% glycerol (*v*/*v*). In all experiments, log-phase cultures of bacteria grown in TSB (or M17 broth supplemented with 0.5% glucose for *L. lactis*) were centrifuged at 3000× *g*, washed three times, and resuspended in phosphate-buffered saline (PBS, pH 7.4). Bacterial titers were determined using optical density measurement and validated by plating on nutrient agar plates.

### 2.2. Biopanning of Phage-Displayed Peptides

The Ph.D-12 Phage Display Peptide Library (New England Biolabs, Ipswich, MA, USA) was screened for *K. pneumoniae*-binding phages as previously described with slight modifications [34]. To ensure specificity, the phage library was depleted of clones binding to bovine serum albumin (BSA)-coated wells before selecting *K.* *pneumoniae*-binding clones. For the first round of biopanning, *K. pneumoniae* KCTC 2208 cells resuspended in PBS (OD_600_ ~ 1) were coated in wells of a high binding microtiter plate (Nunc MaxiSorp, Thermo Fisher Scientific, Rochester, NY, USA) by incubating overnight at 4 °C. After blocking with 5% BSA in tris-buffered saline (TBS, 50 mM Tris-HCl, pH 7.5, 150 mM NaCl), the plates were incubated with the phage library at a final concentration of 1 × 10^11^ (100 μL/well) for 1 h at room temperature. The unbound phages were removed by washing ten times with TBST (TBS containing 0.05% Tween-20), and the bound phages were then eluted by adding 100 μL of glycine-HCl (pH 2.2, 1 mg/mL BSA) for 10 min. The eluted phages were neutralized with 1 M Tris-HCl (15 μL, pH 9.1), amplified, purified, and titrated according to the manufacturer’s instructions. Each subsequent selection round used 1 × 10^11^ phage derived from the phage library retrieved in the previous round. After the sixth round of biopanning, DNA was isolated from the selected phage clones and sequenced at Cosmogenetech (Seoul, Korea). The Snapgene viewer software was used to convert the DNA sequences into amino acids (GSL biotech; available at snapgene.com, accessed on 04 October 2021).

### 2.3. Preparation of Biotinylated Peptide

The peptide used in this study was commercially synthesized at Peptron (Daejeon, Korea) via the Fmoc-based solid-phase peptide synthesis protocol. During the synthesis, the peptide was biotinylated by adding a Lys residue at the COOH terminus with biotin covalently linked to the ε-amine. The biotinylated peptide was prepared to a purity of >95% by reversed-phase high-pressure liquid chromatography (HPLC) using a Capcell Pak C18 column (Shiseido, Tokyo, Japan), and the molecular weight of the biotinylated peptide was confirmed by liquid chromatography/mass spectrometry (LCMS-2020, Shimadzu, Kyoto, Japan).

### 2.4. Phage Binding ELISA Assay

The microtiter plates were coated with bacterial cells (1 × 10^7^ colony-forming unit (CFU)/well) overnight at 4 °C and incubated again with 3% BSA in TBS for 1 h at room temperature to block non-specific binding. After washing three times with TBST, one hundred microliters of amplified phages (1 × 10^9^ plaque-forming unit) were added and incubated for 1 h. The wells were washed three times with TBST; subsequently, horseradish peroxidase (HRP)-conjugated anti-M13 monoclonal antibody (1:5000, GE Healthcare, Chicago, IL, USA) was added and incubated for 1 h. The wells were rewashed with TBST, and 2,2′-azino-bis (3-ethylbenzothiazoline-6-sulfonic acid) (ABTS) peroxidase substrate (Sigma-Aldrich, St. Louis, MO, USA) was added to the wells to detect phage binding. The color development at 415 nm was recorded using a microplate reader (Synergy, BioTeK, Santa Clara, CA, USA).

### 2.5. Binding Specificity of KP Peptide

To analyze the specificity of the peptide identified by phage display, the peptide was subjected to ELISA against different bacteria as described in Section 2.4. The bacterial cells were resuspended in PBS and incubated in ELISA plates overnight at 4 °C to achieve coating. Subsequently, ELISA was performed using the biotinylated KP peptide (10 μM) as the recognition element. The bound peptide was detected using the streptavidin-HRP conjugate (1:2000 in blocking buffer, Thermo Fisher Scientific).

### 2.6. Confocal Laser Scanning Microscopy

Bacterial cells (1 × 10^7^ CFU) in PBS were inoculated on a poly-_L_-lysine coated chamber slide at room temperature for 1 h, then washed three times with TBS. Biotinylated KP peptide (10 μM) was added to the immobilized bacterial cells for 1h, and then slides were rewashed with TBS. The bounded peptide was visualized by applying 5 mg/mL streptavidin-AlexaFluor 488 (Invitrogen, Carlsbad, CA, USA) and observation with an FV1000 confocal laser scanning microscope (Olympus, Tokyo, Japan).

### 2.7. Binding of KP Peptide to Lipopolysaccharide

Lipopolysaccharide (LPS) from *K. pneumoniae* ATCC 15380, *P. aeruginosa* ATCC 27316, *S. typhimurium* ATCC 7823, and lipid A from *Salmonella enterica* serotype minnesota Re 595 were purchased from Sigma-Aldrich. LPS from *E. coli* ATCC 12014 was purchased from InvivoGen (San Diego, CA, USA). LPS was also extracted from *K. pneumoniae* KCTC 2208, *E. coli* KCTC 2223, *P. aeruginosa* ATCC 27853, and *S. typhimurium* KCTC 2370 with the Lipopolysaccharide extraction kit (iNtRON Biotechnology, Seongnam, Korea) according to the manufacturer’s instructions. Two milliliters of bacterial cultures with OD_600_~1 were pelleted by centrifugation. After applying lysis and purification buffers, the LPS pellet was dissolved by boiling for 3 min. The concentration of extracted LPS was determined by comparing it to a standard curve of commercially available LPS using purpald assay [35].

To examine the binding of KP peptide to LPS, each microtiter plate well was coated with 100 μL of LPS (2 μg/well) in 50 mM NaHCO_3_, pH 8.6 overnight at 4 °C. Biotinylated KP peptide (10 μM) or biotinylated polymyxin B (1:50, HycultBiotech, Uden, The Netherlands) was added to the plates. After incubation and washing, the streptavidin-HRP conjugate was added to the plate for detection. The other steps were performed as described above (see detailed protocol in Section 2.4).

### 2.8. K. pneumoniae O Antigen Typing

O typing was performed by using a two-step PCR method described by Fang et al. with minor modifications [36]. Genomic DNA was extracted from 2 mL bacterial solution with a G-spin^TM^ Genomic DNA Extraction Kit (for bacteria) (iNtRON Biotechnology). Using 10 ng of genomic DNA as the template, PCR was performed with GoTaq^®^ G2 Hot Start Green Master Mix (Promega, Madison, WI, USA) and 0.5 μM primers. O1, O2a, O2ac O3, O5, O8, and O9 were determined using two primer pairs for each allele at the wb gene cluster. A genotype is assigned only to the strains that tested positive for both primer pairs. The strains bearing the O1/O2 allele were examined again with the second set of PCR primers (O1- and O2ac-specific primers). The following cycling conditions were used: 95 °C for 2 min, then 30 cycles of 95 °C for 1 min, 56 °C for 1 min, and 72 °C for 2 min, followed by a 5 min extension at 72 °C. PCR product size was determined on a 1.5% agarose gel. The primer sequences and PCR product size are shown in Table 1.

### 2.9. Comparison of Specificity and Sensitivity of KP Peptide with Commercially Available Anti-Klebsiella spp. Antibodies

Ten-fold serial dilutions of log-phase bacterial cultures were added to wells of a microtiter plate and incubated overnight at 4 °C. Biotinylated KP peptide (10 μM) and two commercially available biotinylated anti-*Klebsiella* spp. polyclonal antibodies (1:5000, GTX36396 (Genetex, Irvine, CA, USA), ab69468 (Abcam, Cambridge, UK)) were added to the plate. After incubation and washing, the streptavidin-HRP conjugate was added to the plate for detection. The other steps were performed as described above (see detailed protocol in Section 2.4).

## 3. Results and Discussion

### 3.1. Biopanning of a Phage Display Library against K. pneumoniae

The one-bead, one-compound (OBOC) combinatorial peptide library and the phage-displayed peptide library are the two most popular approaches that have been successfully used for the discovery of novel peptide ligands [37,38]. The OBOC strategy allows for the use of building blocks other than natural amino acids such as _D_-amino acids, which can confer protease resistance [39,40], but OBOC screening is technically difficult and typically examines only ~10^6^ compounds [41,42]. The main advantage of the phage-displayed peptide library is that it can generate a library with higher sequence diversity (10^9^ for phage-displayed library vs. 10^6^ for OBOC library), which is critical for the selection and screening of high-affinity peptides. Because of its unparalleled diversity and commercial availability, we used a phage-displayed random peptide library to isolate clones displaying peptides that can bind specifically to the whole *K. pneumoniae* cell surface. After eliminating the phages that interacted with BSA, *K. pneumoniae* was probed by the remaining phages in the peptide library. After washing to remove the unbound phages, *K. pneumoniae*-binding phages were eluted, propagated, and purified for input in the next round of biopanning. After six rounds of biopanning, 24 phage clones were randomly selected from the eluted phage population and amplified. Genomic DNA was extracted from each amplified phage and sequenced to determine the peptide sequence displayed in the major coat protein. All 24 clones were found to encode the same peptide sequence (TSATKFMMNLSP).

### 3.2. Specificity of Phage Binding to K. pneumoniae

**Figure 1:**
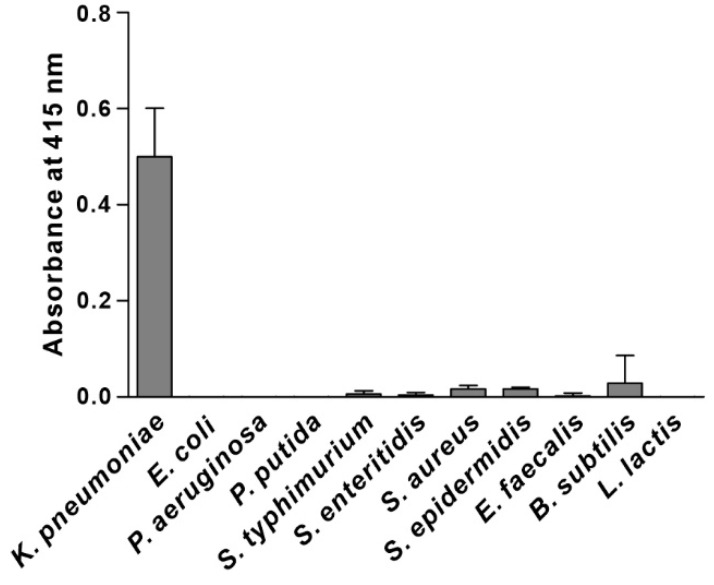
Specificity and selectivity of phage clone binding to *K. pneumoniae*. The binding of the selected phage clone to different bacterial cells was determined by phage-binding ELISA. Microtiter plates were coated with bacterial cells, incubated with the selected phage clone, and then incubated with HRP-conjugated anti-M13 antibody. The substrate ABTS was used to detect the positive binding of the phage clone to bacteria. The data are presented as the mean ± SD of three independent experiments performed in triplicate.

Specificity is the ability of a recognition element to interact preferentially with the target over other potential ones. To determine the recognition specificity, we examined the ability of the selected phage clone to interact preferentially with *K. pneumoniae* compared to other possible targets, including Gram-negative bacteria (*E. coli*, *P. aeruginosa*, *P. putida*, *S. typhimurium*, and *S. enteritidis*) and Gram-positive bacteria (*S. aureus*, *S. epidermidis*, *E. faecalis*, *B. subtilis*, and *L. lactis*). The results shown in Figure 1 suggest that the selected phage clone bound specifically to *K. pneumoniae* and did not cross-react with other species. Hence, the peptide sequence displayed in the major coat protein of the selected phage clone (TSATKFMMNLSP, named KP peptide) was synthesized and further tested for its suitability as a recognition element for *K. pneumoniae* detection.

### 3.3. Specificity of KP Peptide Binding to K. pneumoniae

**Figure 2:**
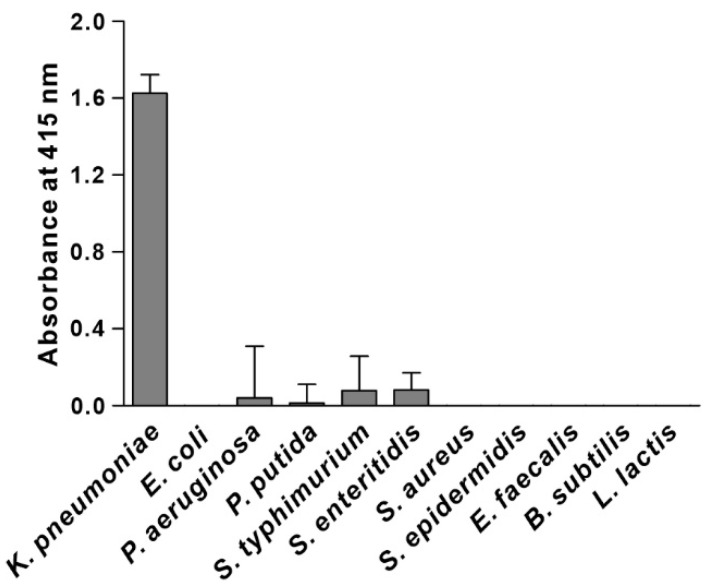
Specificity and selectivity of KP peptide binding to *K. pneumoniae.* The binding activity of KP peptide to different bacterial cells was determined by peptide-binding ELISA. Microtiter plates were coated with bacterial cells and incubated with the biotinylated KP peptide. The bound peptide was detected by HRP-conjugated streptavidin and the substrate ABTS. The data are presented as the mean ± SD of three independent experiments performed in triplicate.

**Figure 3:**
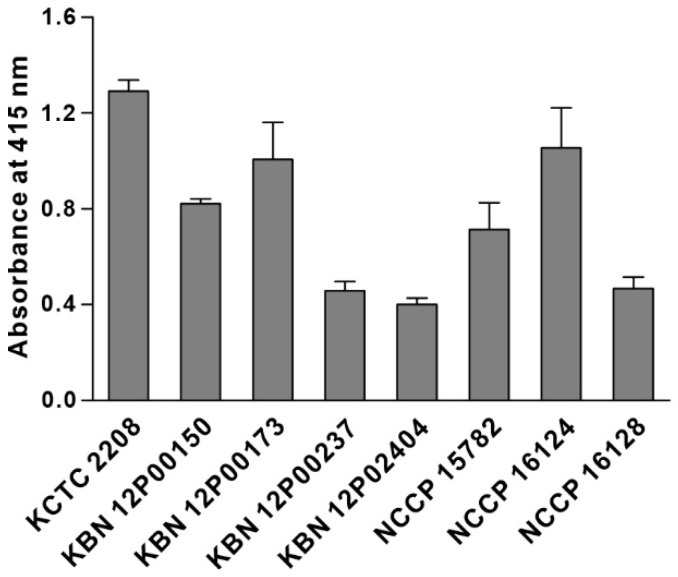
Capacity of KP peptide to detect clinical *K. pneumoniae* isolates. Microtiter plates were coated with *K. pneumoniae* KCTC 2208 and seven clinical isolates and incubated with the biotinylated KP peptide. The bound peptide was detected by HRP-conjugated streptavidin and the substrate ABTS. The data are presented as the mean ± SD of three independent experiments performed in triplicate.

**Figure 4:**
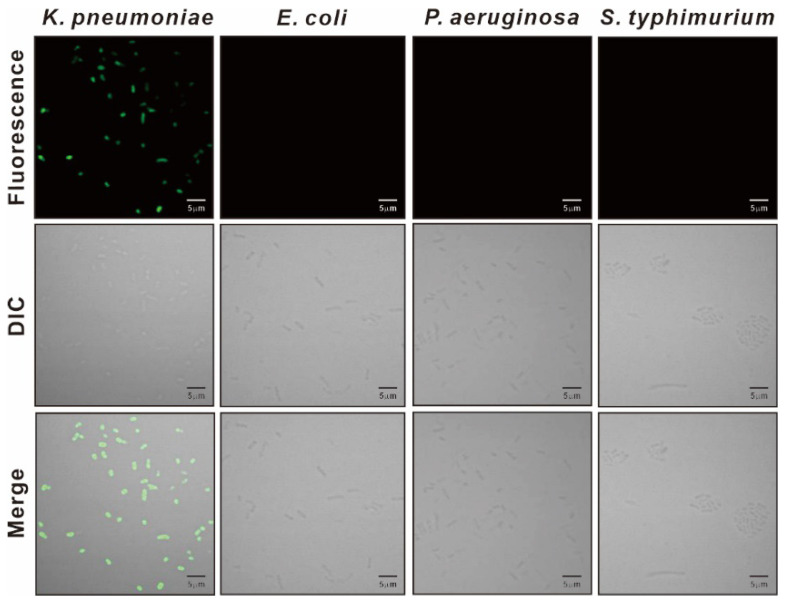
Confocal laser scanning microscopy of bacteria treated with KP peptide. Bacterial cells were incubated with the biotinylated KP peptide and visualized with Alexa 488-labeled streptavidin.

We synthesized the KP peptide with a C-terminal Lys as a biotin tag and investigated the ability of this peptide to interact preferentially with *K. pneumoniae* relative to a panel of selected Gram-negative bacteria and Gram-positive bacteria. As illustrated in Figure 2, KP peptide showed significant binding to *K. pneumoniae* and little or no binding to the other bacterial strains as observed with the phage-binding ELISA. We then assessed the binding capacity of KP peptide against seven clinically isolated *K. pneumoniae* strains, including antibiotic-resistant strains (Figure 3). ELISA assay confirmed that KP peptide is a specific and selective recognition element for different *K. pneumoniae* strains, including the antibiotic-resistant strains. As the KP peptide showed specific binding to *K. pneumoniae* in ELISA, we further investigated its ability to bind to the target bacterium using confocal laser scanning microscopy. Figure 4 demonstrates that the peptide was explicitly bound to the outer surface of *K. pneumoniae*, supporting the ELISA results.

### 3.4. Characterization of the Binding Specificity of KP Peptide

**Figure 5:**
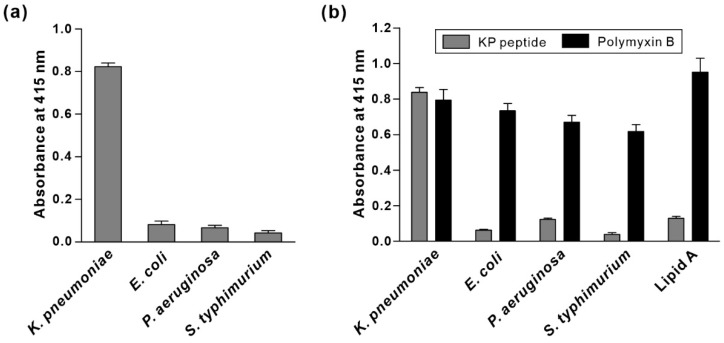
Validation of binding activity of KP peptide to LPS. The binding of KP peptide to LPS from *K. pneumoniae* and their cross binding to LPS from the other Gram-negative bacteria were determined by peptide-binding ELISA. Extracted LPS (**a**), commercially available LPS, and lipid A (**b**) were coated onto microtiter plates. Peptides (biotinylated KP peptide and polymyxin B) bound to the coated LPS were detected by HRP-conjugated streptavidin and the substrate ABTS. The data are presented as the mean ± SD of three independent experiments performed in triplicate.

To identify potential binding targets for the KP peptide on the surface of *K. pneumoniae*, we first checked whether KP peptide binds to outer membrane proteins on *K. pneumoniae*. Total outer membrane proteins (OMPs) of *K. pneumoniae* were isolated, and far-western blotting analysis and pull-down assay were performed by using biotinylated KP peptide as a probe, as previously described [34]. For far-western blotting, total OMPs were separated by SDS-PAGE, blotted to a PVDF membrane and probed with biotinylated KP peptide. For pull-down assay, OMPs and biotinylated KP peptide were mixed and pulled down through conjugation with streptavidin-coupled magnetic beads. The captured proteins were separated and visualized by Coomassie blue staining. We could not detect any band in both assays, suggesting that KP peptide did not bind to outer membrane proteins on *K. pneumoniae* (data not shown). Next, we investigated the ability of the KP peptide to interact with LPS, which is the major component of the outer membrane of Gram-negative bacteria. LPS was extracted from various Gram-negative bacteria, and its association with KP peptide was examined by ELISA. As shown in Figure 5a, KP peptide strongly bound to LPS from *K. pneumoniae* compared with LPS from other bacteria. To verify the finding that KP peptide bound to *K. pneumoniae* LPS, KP peptide binding to the commercially available LPS from different bacterial strains was investigated. We also included lipid A in the assay to observe whether KP peptide can bind to the highly conserved lipid A portion of LPS. Polymyxin B was used as a positive control due to its strong binding capacity for lipid A. As shown in Figure 5b, polymyxin B displayed a high affinity towards all the tested LPS, with the maximum affinity for lipid A. In contrast to polymyxin B, KP peptide bound to a great extent to LPS derived from *K. pneumoniae*, whereas little binding was observed for lipid A and LPS derived from other bacteria. Overall, these data indicated that KP peptide binds specifically to LPS on *K. pneumoniae,* and this specificity might not be the result of the binding to lipid A.

**Figure 6:**
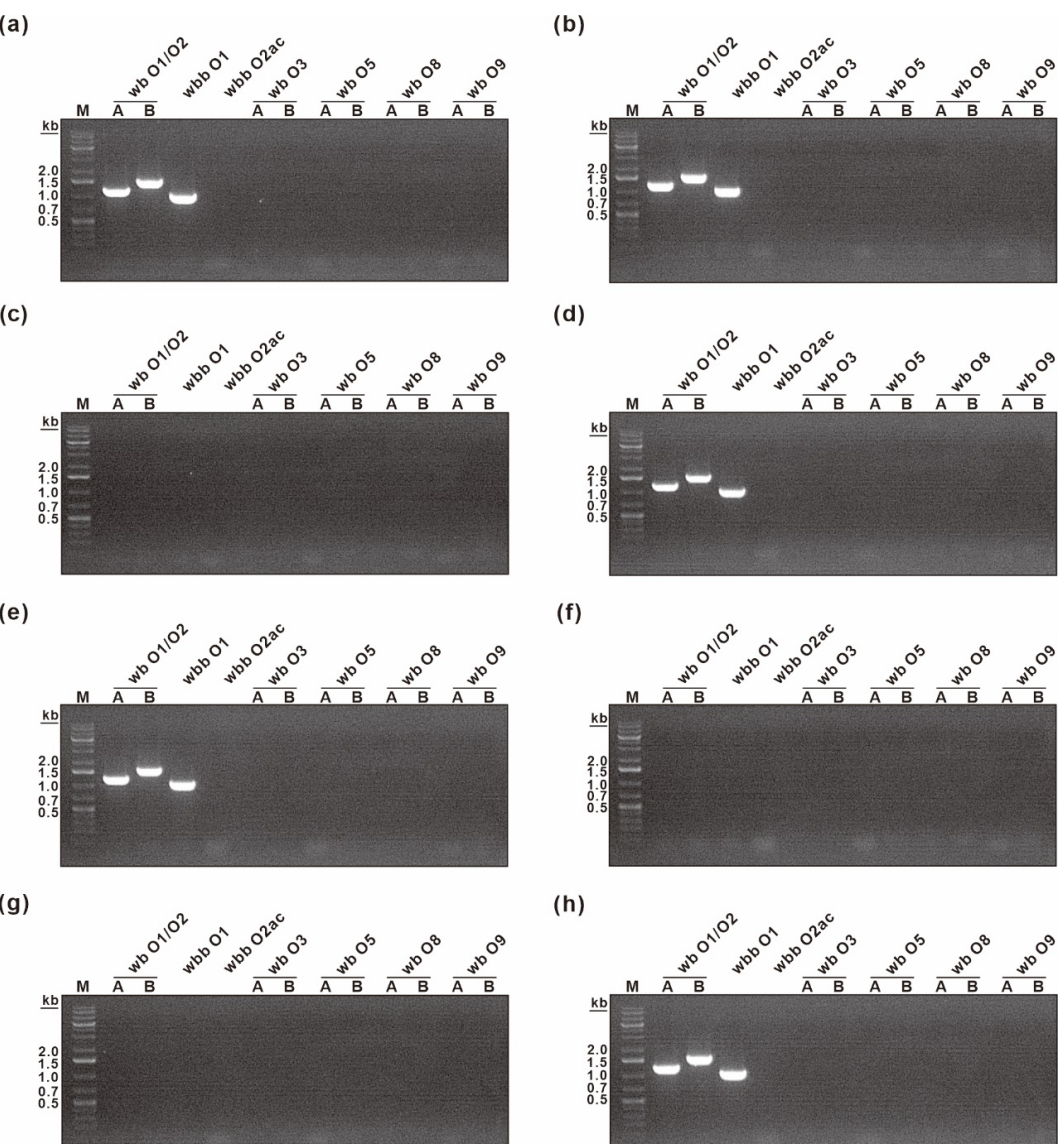
Genotyping of O antigen in a panel of clinical *K. pneumoniae* isolates by PCR. Agarose gel images showing the PCR amplification of the primers using the genomic DNA of *K. pneumoniae* as the template were presented. The primer pair name was indicated on top of each lane. The band sizes of the DNA ladder are shown on the left. (**a**) *K. pneumoniae* KCTC 2208, (**b**) *K. pneumoniae* KBN 12P00150, (**c**) *K. pneumoniae* KBN 12P00173, (**d**) *K. pneumoniae* KBN 12P00237, (**e**) *K. pneumoniae* KBN 12P02404, (**f**) *K. pneumoniae* NCCP 15782 (**g**) *K. pneumoniae* NCCP 16124, (**h**) *K. pneumoniae* NCCP 16128.

As in other *Enterobacteriaceae*, three domains are recognized in the *K. pneumoniae* LPS: the highly conserved lipid A, the highly variable O antigen polysaccharide, and the core oligosaccharide connecting lipid A and O antigen. To determine to which part of the LPS the KP peptide binds specifically, we performed O typing on eight *K. pneumoniae* strains, which were used in the ELISA binding assay in Figure 3. The O antigen differs significantly between *K. pneumoniae* strains based on the sugar residues and their linkage patterns within the repeating subunits and is classified into 11 serotypes: O1, O2a, O2ac, O2afg, O2aeh (O9), O3, O4, O5, O7, O8, and O12 [43]. When the galactose-based polysaccharides (O1, O2, O8, and O9 serotype) and mannose-based polysaccharides (O3 and O5 serotype) were determined, the results showed that five *K. pneumoniae* strains (KCTC 2208, KBN 12P00150, KBN 12P00237, KBN 12P02404, and NCCP 16128) belonged to the O1 serotype, while the remaining three strains belonged to other O serotypes (i.e., they did not belong to O1, O2, O3, O5, O8, and O9) (Figure 6). These results suggested that O antigen is not the main target of KP peptide because KP peptide could bind to multiple *K. pneumoniae* strains with different O antigens, as shown in Figure 3. In contrast to O antigens, two types of core oligosaccharides were described in *K. pneumoniae*; both share the same inner core and outer core proximal disaccharide with differences in the glucosamine substituents [44,45]. The inner core of *K. pneumoniae* differs from those of *E. coli* and *Salmonella*, though the inner core is highly conserved within the *Enterobacteriaceae* [45,46]. We thus hypothesized that the specificity of the KP peptide is due to its interaction with the core oligosaccharide region of *K. pneumoniae* LPS. However, additional research is required to validate our hypothesis.

### 3.5. Comparison of Specificity and Sensitivity of KP Peptide with Commercially Available Anti-Klebsiella spp. Antibodies

**Figure 7:**
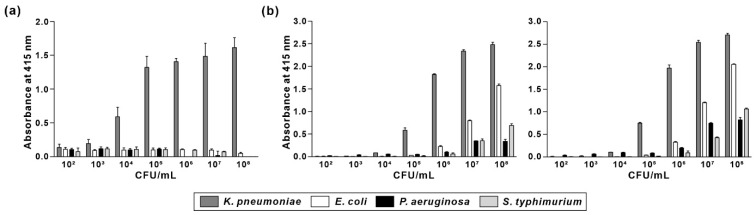
Comparison of specificity and sensitivity of KP peptide with commercially available anti-*Klebsiella* spp. antibodies. Microtiter plates were coated with different concentrations of bacterial cells (10^2^–10^8^ CFU/mL) and incubated with biotinylated KP peptide (**a**) or anti-*Klebsiella* spp. antibodies [(**b**) left: ab69468; right: GTX36396] prior to incubation with streptavidin-HRP. The substrate ABTS was used to detect the positive binding of the peptide or antibodies to bacteria. The data are presented as the mean ± SD of three independent experiments performed in triplicate.

To evaluate the suitability of KP peptide as a recognition element for the detection of *K. pneumoniae*, specificity and sensitivity were compared with that of commercially available anti-*Klebsiella* spp. antibodies. Biotinylated KP peptide or antibodies were incubated with pre-immobilized Gram-negative bacterial cells (from 10^2^ to 10^8^ CFU/mL), and the binding interaction was measured using ELISA. As shown in Figure 7, anti-*Klebsiella* spp. antibodies showed specific binding for *K. pneumoniae* at bacterial concentrations ranging from 10^5^ to 10^6^ CFU/mL. However, their specificity for *K. pneumoniae* was decreased as the number of bacterial cells was increased from 10^7^ to 10^8^ CFU/mL. In contrast, KP peptide showed good specificity for *K. pneumoniae* at all the tested bacterial concentrations compared to that for other Gram-negative bacteria. Moreover, the detection limit of KP peptide was 10^4^ CFU/mL, which was ten times more sensitive than anti-*Klebsiella* spp. antibodies. These data indicated that KP peptide is more specific and sensitive than anti-*Klebsiella* spp. antibodies as a recognition element for detecting *K. pneumoniae*.

Because ELISA is not suitable for pathogen detection at the point-of-care, proper detection methods should be developed in the future to use this peptide as a recognition element for a biosensor analytical device for *K. pneumoniae* detection at the point-of-care. As a common strategy to improve the sensitivity and accuracy of detection methods, the preconcentration to the low-abundant analytes can be used. Immunomagnetic nanoparticles, which can enrich the cells rapidly without bacterial cultivation and centrifugation, might be used for the detection method [47,48]. Future work will focus on the development of a KP peptide-based biosensor for *K. pneumoniae* detection.

## 4. Conclusions

The discovery of novel peptide sequences that specifically bind to the target bacteria can be regarded as a first step toward the development of a bacterial detection system. In this study, we identified a unique short peptide (KP peptide, TSATKFMMNLSP) specific to *K. pneumoniae* cells from the biopanning of the M13 phage display peptide library and measured the binding interactions of the peptide-displayed phage clone and the synthetic peptide using ELISA. KP peptide was specific to *K. pneumoniae* and had low cross-reactivity with other Gram-negative bacteria. The selectivity of the KP peptide against *K. pneumoniae* resulted from the specific interaction between the KP peptide and *K. pneumoniae* LPS. Furthermore, KP peptide possessed higher specificity and sensitivity toward *K. pneumoniae* than commercially available anti-*Klebsiella* spp. antibodies. This peptide that selectively binds to and detects *K. pneumoniae* might be a cost-effective alternative to antibodies and a useful diagnostic tool for the detection of *K. pneumoniae*.

## Figures and Tables

**Table 1:** Primers used for *K. pneumoniae* LPS O genotyping by PCR.

O Type Detected	Primer	Sequence (5′–3′)	PCR Product Size (bp)
O1/O2	wb O1/O2-A-F	CGCTATAAGAGCAGCATGCTAG	1251
	wb O1/O2-A-R	CGATATCACCTACTGCCAGA	
	wb O1/O2-B-F	TTGTTGAGCCTGACAGGATC	1589
	wb O1/O2-B-R	GCCATTGCTTGCTTGTACAG	
O1	wbb O1-F	GATTTCACTTTCCGGGCAAC	1075
	wbb O1-R	GGCTTGCTGAATCACAAGAC	
O2ac	wbb O2ac-F	AAACATCGCTGACTCGAGTC	1046
	wbb O2ac-R	CGACTATGATCGTACCAACG	
O3	wb O3-A-F	CTATCGCTACCGTGGCTTTA	767
	wb O3-A-R	TCTCGTCCACAATATCAGCG	
	wb O3-B-F	GCCTACAGTATCTACCTCTG	903
	wb O3-B-R	CGGTAAAGTCAGGATGGAAG	
O5	wb O5-A-F	GCTACCAAACCAGTATGCTG	1821
	wb O5-A-R	AGGTGCGTACTGGAAGTATG	
	wb O5-B-F	GGTGATGAAAGCCAGAATGC	1423
	wb O5-B-R	CAGTGCCTGAAACAGTTTGC	
O8	wb O8-A-F	CGTGGCAATGGTTTGCTAGT	1230
	wb O8-A-R	TCAATCCACACAACTCGGTC	
	wb O8-B-F	GCTAGTTCGGCAACTAACTCAC	841
	wb O8-B-R	AGTTCCAGCATCGAAGCAACTC	
O9	wb O9-A-F	CGCGCTCAGTTATTCCATTG	973
	wb O9-A-R	CTGGCTGATGACAGAGAATC	
	wb O9-B-F	GCATTCCTGTTCGTGTATGG	949
	wb O9-B-R	ATGTCACCGACAGCAAGTAC	

## Data Availability

Not applicable.
